# Supplementary material for: psiCLIP reveals dynamic RNA binding by DEAH-box helicases before and after exon ligation
Source: Nat Commun. 2021 Mar 5;12:1488. doi: 10.1038/s41467-021-21745-9 (PMC7935899; doi:10.1038/s41467-021-21745-9)
Supplement: Supplementary file 1 — Supplementary Information [file 41467_2021_21745_MOESM1_ESM.pdf]

Supplementary Information

psiCLIP reveals dynamic RNA binding by DEAH-box helicases before and after exon ligation

Lisa M. Strittmatter[1*], Charlotte Capitanchik[2*], Andrew J. Newman[1], Martina Hallegger[2,3], Christine M. Norman[1], Sebastian M. Fica[1,†], Chris Oubridge[1], Nicholas M. Luscombe[2,4,5,†], Jernej Ule[2,3,§,†], and Kiyoshi Nagai[1]

* These authors contributed equally. † Joint corresponding authors
§ Lead contact
1.MRC Laboratory of Molecular Biology, Cambridge CB2 0QH, UK
2.The Francis Crick Institute, London NW1 1AT, UK
3.Department of Neuromuscular Diseases, UCL Queen Square Institute of Neurology, Queen Square, London WC1N 3BG, UK
4.UCL Genetics Institute, Department of Genetics, Environment and Evolution, University College London, London WC1E 6BT, UK
5.Okinawa Institute of Science & Technology Graduate University, Okinawa 904-0495, Japan

Supplementary Figures

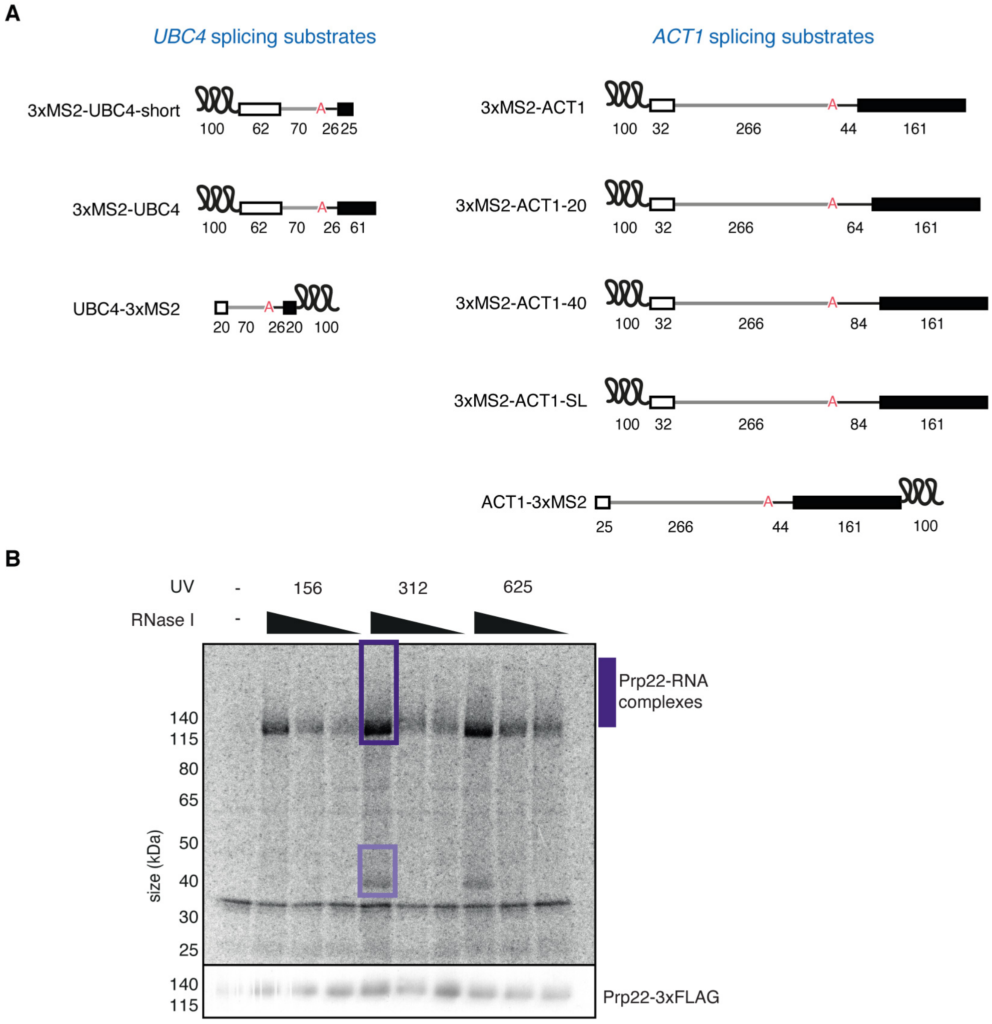

Supplementary Fig. 1 Splicing substrates used in psiCLIP. A All transcripts were generated to include 3 MS2 stem loops for spliceosome purification. The transcripts are derived from the budding yeast *UBC4* (left) and *ACT1* genes (right). Distances between the splice sites are given in nucleotides. **B** Autoradiograph for parallel fine-tuning of UV-irradiation and RNase I digest (top panel). Purified complex C* containing endogenous Prp22-3xFLAG was irradiated with the indicated doses of UV (in 100 x µJ/cm2), and fragmented by the addition of 5, 0.5 or 0.05 units of RNase I following the psiCLIP procedure as described in the methods section. Bottom panel shows subsequent Western probing with anti-FLAG antibody to visualise Prp22 protein levels. The dark purple box indicates the membrane area selected for cDNA library preparation from the optimised condition for Prp22 crosslinking. The light purple box shows a co-crosslinked protein that was released with the given RNase I concentration. A high RNase concentration was optimal for liberating a multi-protein crosslink (light purple box).

Supplementary Figures

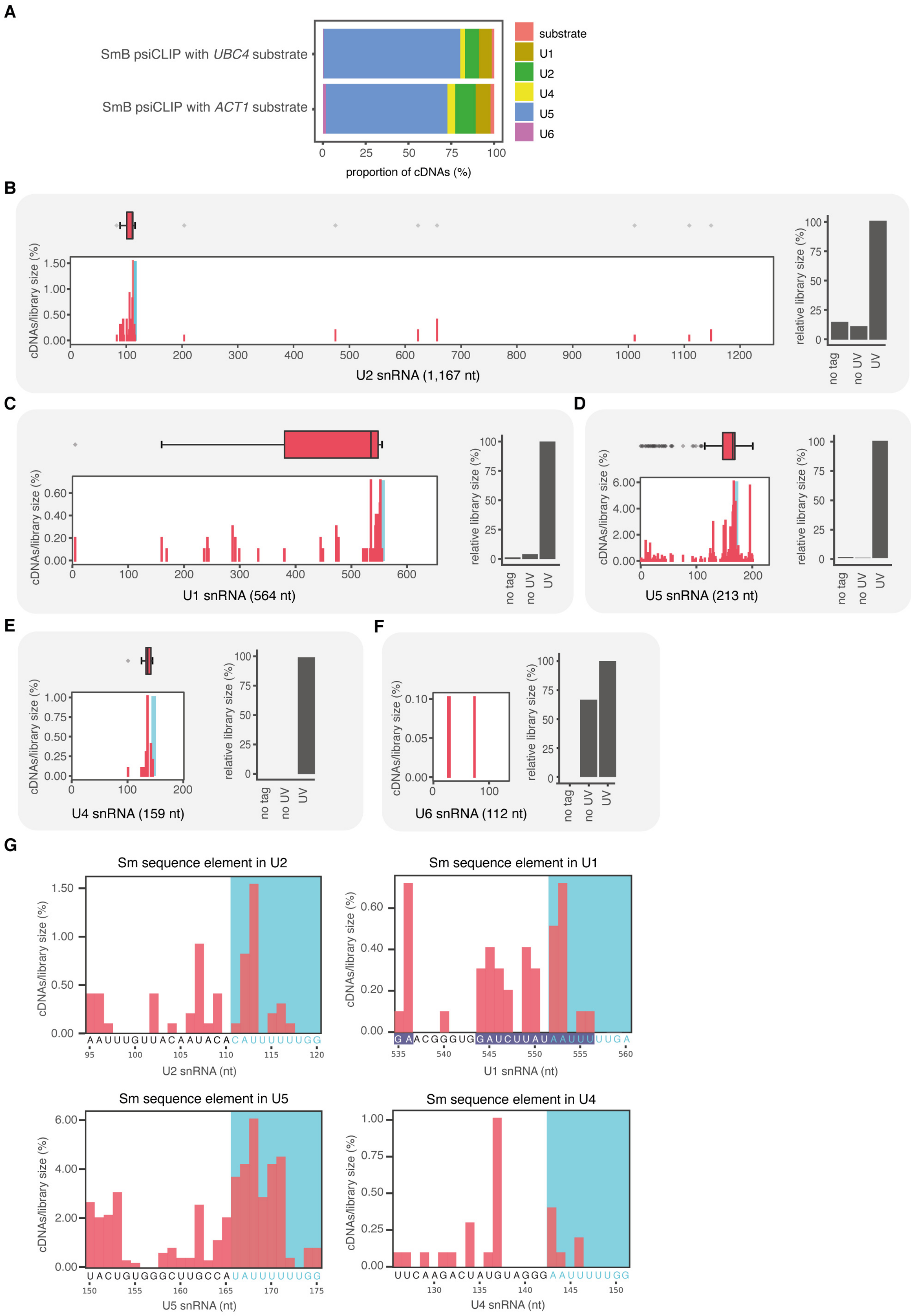

Supplementary Fig. 2 Validation of the positional specificity of psiCLIP with SmB. A Proportion of cDNAs mapping to substrate and snRNAs for two SmB psiCLIP experiments. **B-F** Crosslinking to snRNAs from experiment that used the short *UBC4* substrate. Crosslink events (-1 position of cDNA start) are represented as histograms and box plots aligned to the indicated snRNA. On the histograms, the y-axis indicates the proportion of cDNAs out of all cDNAs mapped to snRNAs and pre-mRNA substrate. The box plots represent crosslink positions from one sample along the transcript, weighted by normalised cDNA count. The line across the box represents the median, the lower and upper bounds correspond to the first and third quartiles. The whiskers end at the largest and smallest value no further than 1.5 times the inter-quartile range. Outliers outside of this range are plotted as dots. The bar charts represent the proportion of cDNAs in samples, normalised to the UV condition, which is shown as 100%. No tag: UV irradiated untagged SmB, No UV: SmB-3xFLAG without UV irradiation, UV: UV irradiated SmB-3xFLAG. As expected, very few crosslinks are found on the U6 snRNA, which is bound by the LSm ring that lacks the SmB protein; due to very low crosslinking on U6 snRNA the box plot is not shown. **G** Zoom in to show crosslinking at the Sm sequence elements, which are highlighted in blue letters and blue background. The U1 snRNA region highlighted in purple is shown in Fig 2D, mapped onto the cryo-EM structure.

Supplementary Figures

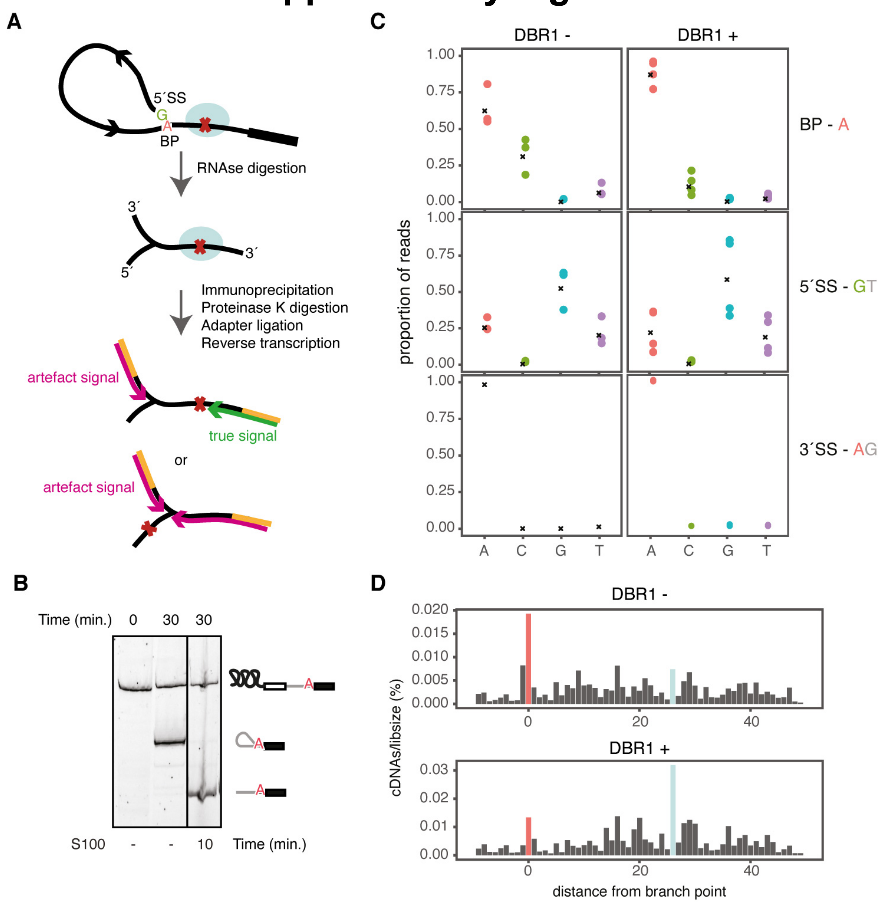

Supplementary Fig. 3 DBR1 treatment resolves intron-lariats. A A schematic showing how intron-lariats can cause artefactual signal. If a protein (in blue) crosslinks near to the branch point, upon RNase digestion part of the lariat may from a Y-shaped structure. In this situation, the free 3′ end of the lariat is available for adaptor ligation (in orange). Thus the final library can have many reads that begin at the 5′-SS and branch-point and which originate from the lariat-intermediate and not from a protein-RNA crosslink. **B** Treatment of lariats with S100 cell lysate fraction leads to debranching. Intron-lariat intermediates were generated by *in vitro* splicing reactions of 3xMS2-UBC4-AC (lane 2). Treatment with S100 for 10 min in debranching buffer leads to full debranching. Only splicing products containing the second exon are visible as the 3′ end of the transcript is fluorescently labelled. This experiment was repeated three times (n=3) with different incubation times for debranching of 10min, 30min and 60min; a similar effect was observed for all experiments. **C** Reverse transcription of the 5′-SS guanosine and branch-point adenosine can result in mutation. In the final reads, the branch-point nucleotide is often read as a cytosine (top) and the 5′-SS guanosine can be read as an adenosine or thymine (middle). For comparison, the 3′-SS adenosine is rarely mutated (bottom). DBR1 treatment reduces the incidence of mutations (right). Three samples of DBR1- Prp16 psiCLIP and four samples of DBR1+ Prp16 psiCLIP are shown as individual data points. **D** Prp16 crosslinks in a DBR1- and DBR1+ sample, shown normalised to library size. The ratio between the artefact signal (branch-point A in red) and true signal (a representative crosslink shown in blue) shifts dramatically upon DBR1 treatment.

Supplementary Figures

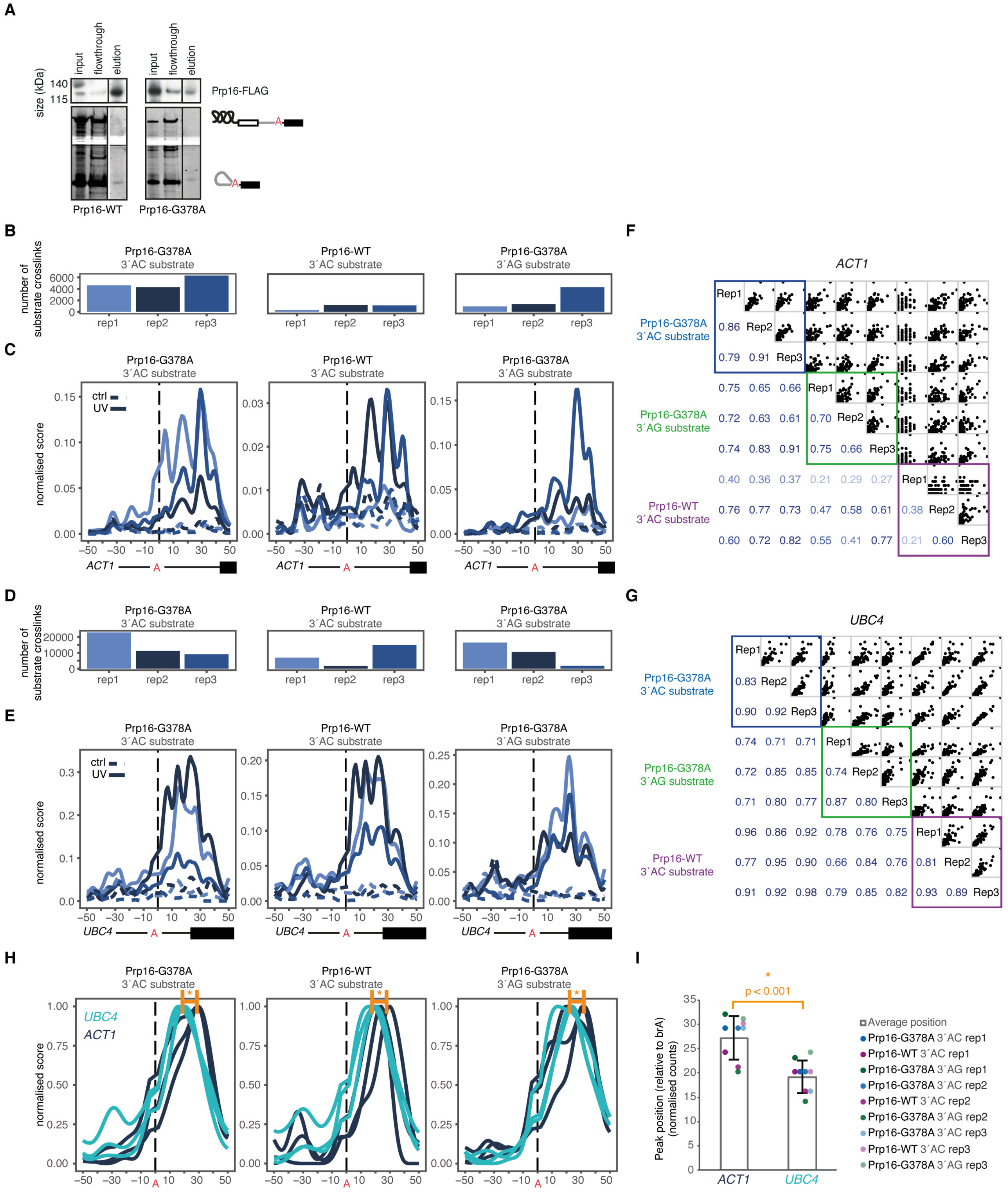

Supplementary Fig. 4 Prp16 psiCLIP data show substrate specific binding in a region downstream of the brA. A Western blot (top) probing the FLAG tag shows Prp16 levels at various steps of the psiCLIP procedure for spliceosomes assembled on *UBC4-3′UAC* and purified via the MS2-tag on the splicing substrate. In this experiment, the sample was not crosslinked to allow protein and RNA to run independently on the gel. Before membrane transfer the gel was scanned for the fluorescently labelled splicing substrate (bottom) and shows that Prp16 binds to lariat-intermediate but not pre-mRNA. Western blots were performed once for each sample. **B** Total number of crosslinks on the *ACT1* pre-mRNA substrate for three replicates of Prp16 psiCLIP. "3′AC" and "3′AG" denote mutant and canonical 3′-SS respectively. **C** Mapping of Prp16 psiCLIP data onto *ACT1*. Positions along the transcript are shown relative to the brA. The smoothed lines show the truncation events normalised to the number of cDNAs mapping to the yeast genome. Lines were Gaussian smoothed with a window size of 10 nucleotides. **D** Same as **A** but for three replicates of *UBC4* Prp16 psiCLIP. **E** Same as **B** but for three replicates of *UBC4* Prp16 psiCLIP. **F-G** Correlation analysis between replicates of Prp16 psiCLIP. The data were normalised to the maximum signal across the substrate so that reproducibility of positional enrichment, rather than crosslinking intensity, could be compared. Each dot represents the normalised crosslinking signal at each position along the substrate. The numbers are Pearson correlation coefficients. Rep1, replicate 1; rep2, replicate 2; rep3, replicate 3. **H-I** Summary of reads normalised to the maximum peak in each sample. Note significant difference between average *ACT1* and *UBC4* peaks, as determined by a two-sided unpaired t-test ($\bar{x}_1 - \bar{x}_2=8$ nucleotides, 95% CI [4, 12], $\alpha=0.05$, d.f.=16, $t_\alpha=4.35$, $p<0.001$). Peak positions for individual replicates are shown in **I**.

Supplementary Figures

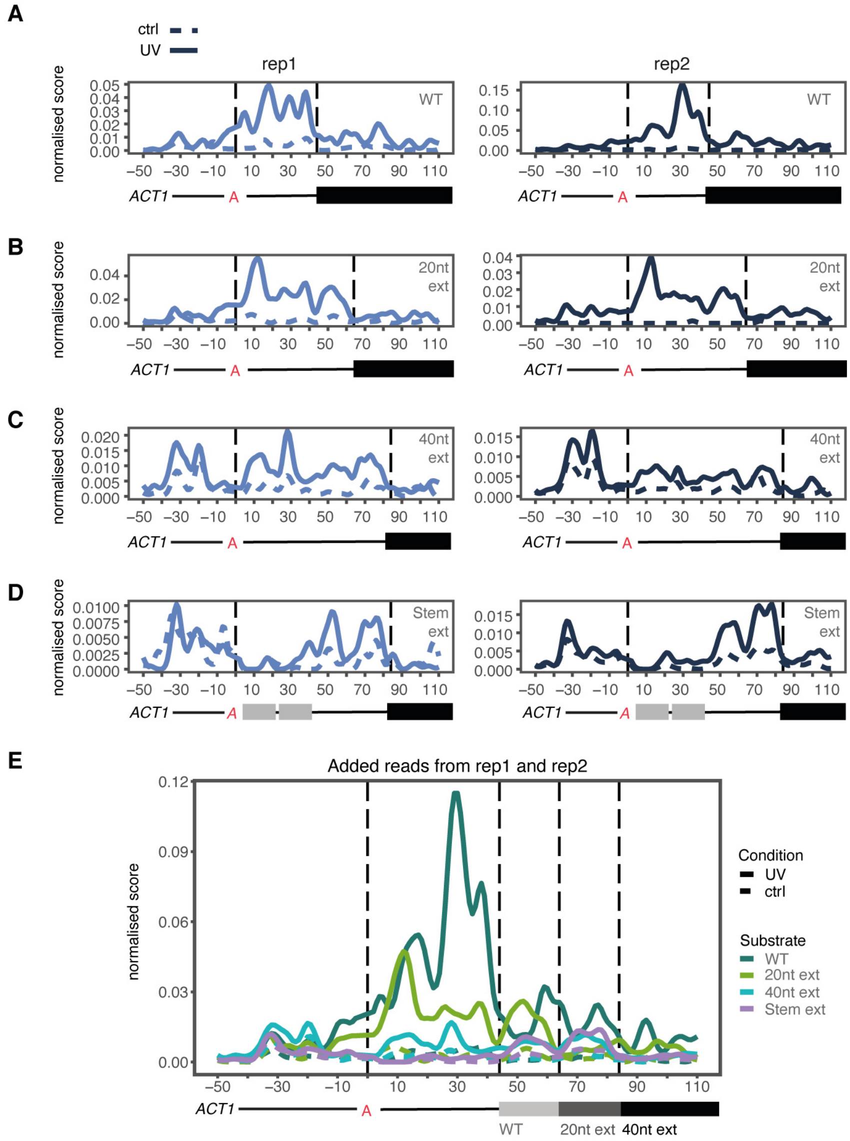

Supplementary Fig. 5 Prp16 binding spans the entire available distance between brA and the 3′-SS. Two replicates for each condition are shown. Mapping of Prp16 mutant psiCLIP data onto the respective *ACT1* splicing substrate. cDNAs are normalised to total cDNAs mapping to the yeast genome. Crosslinks are shown after Gaussian smoothing with a 10 nucleotide window. Positions on the substrate are given relative to brA. **A** Prp16-G378A on the wild-type *ACT1* substrate. **B** Prp16-G378A on *ACT1* substrate with a 20 nucleotide extension between brA and the 3′-SS. **C** Prp16-G378A on *ACT1* substrate with a 40 nucleotide extension between brA and the 3′-SS. **D** Prp16-G378A on *ACT1* substrate with a stem loop in the 40 nucleotide extension between brA and the 3′-SS. The stem-loop region is indicated in grey underneath the graph. **E** The two replicates are summed for each condition, as shown in the main figure, and are shown here on the same scale for comparison. Prp16-G378A binding is reduced on the extended substrates. A sharp decrease in signal from intron to exon for all substrates indicates that the extension of signal observed over the intron results from genuine binding events.

Supplementary Figures

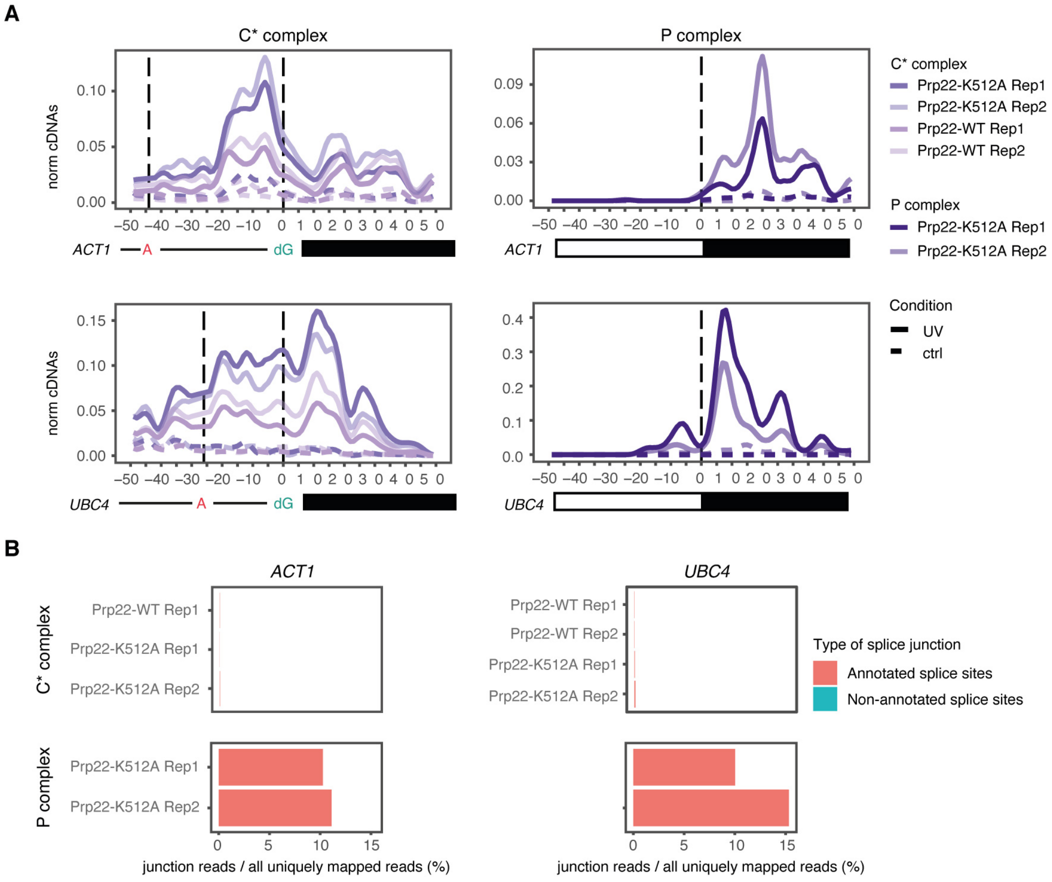

Supplementary Fig. 6 The data pattern for psiCLIP of Prp22 in C* and P complex is consistent between replicates. A Two replicates for each condition are shown. Mapping of Prp22 (WT and mutant) psiCLIP data onto the respective splicing substrate. cDNAs are normalised to total cDNAs mapping to the yeast genome. Crosslinks are shown after Gaussian smoothing with a 10 nucleotide window. Positions on the substrate are given relative to the 3′-SS. P complex crosslinks are shown with the intron removed. Solid lines are tagged experimental conditions and dashed lines are untagged controls. Rep1, replicate 1; rep2, replicate 2. **B** The proportion of spliced reads in each library, showing that C* complex libraries have very few spliced reads, whereas P complex libraries are enriched for spliced reads. The colour denotes whether the splice junction occurs at an annotated, canonical splice site, or a non-annotated, cryptic site. The lack of reads resulting from non-annotated sites indicates that the spliceosome is using the canonical splice sites, as expected in the P complex data.

Supplementary Figures

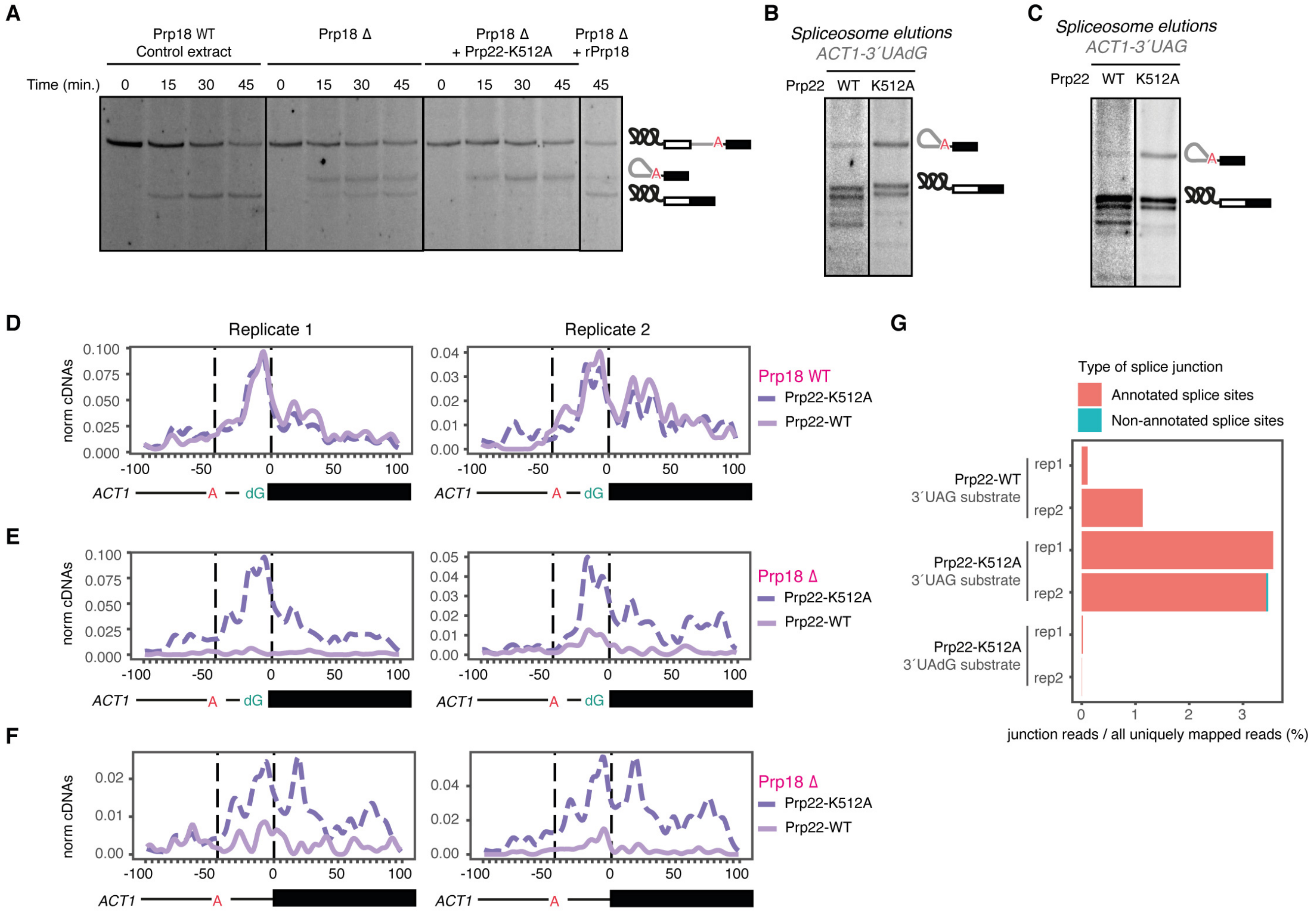

Supplementary Fig. 7 The psiCLIP profile for Prp22 binding following immunodepletion of Prp18 is consistent between replicates. **A** Denaturing gel after splicing assays with 3xMS2-*UBC4* demonstrates that there is a step two splicing defect following depletion of Prp18 both in the background of Prp22-WT protein or with the addition of the dominant negative Prp22-K215A. The second step splicing defect can be rescued after 45 minutes by *in vitro* addition of recombinant Prp18 and is shown with the Prp22 WT background. Prp18Δ denotes extracts depleted of Prp18. **B** Substrate composition of eluted spliceosomes assembled on the 3′-UAdG substrate following depletion of Prp18. Denaturing gel of the spliceosomes eluted from amylose column showing the input for UV-crosslinking and immunoprecipitation. The amount of spliceosomes of the first step are reduced for Prp22 WT background compared to Prp22-K512A. Gels in **A-C** are representative of two independent depletion experiments (n=2). **C** Substrate composition of eluted spliceosomes assembled on the canonical 3′-UAG substrate following depletion of Prp18. Denaturing gel of the spliceosomes eluted from amylose column showing the input for UV-crosslinking and immunoprecipitation. The amount of spliceosomes of the first step are reduced for Prp22 WT background compared to Prp22-K512A. **D-F** psiCLIP of Prp22-WT and Prp22-K512A in spliceosomes assembled on wild-type and 3′-UAdG *ACT1* substrates in wild-type extracts or in extracts depleted of Prp18. Crosslinks are shown as normalised to the total yeast genome mapping reads and with the untagged ctrl subtracted from the tagged UV condition, after Gaussian smoothing with a 10 nucleotide window. **G** The proportion of spliced reads in each Prp18 depletion library, showing that the wild-type substrate can be spliced to make mRNA and this occurs to a higher extent with the Prp22-K512A mutant. The colour denotes whether the splice junction occurs at an annotated, canonical splice site, or a non-annotated, cryptic site. Only a small proportion of reads occurs at non-annotated sites, indicating that the majority of splicing observed occurred at the canonical splice junctions. Rep1, replicate 1; rep2, replicate 2.

Supplementary Figures

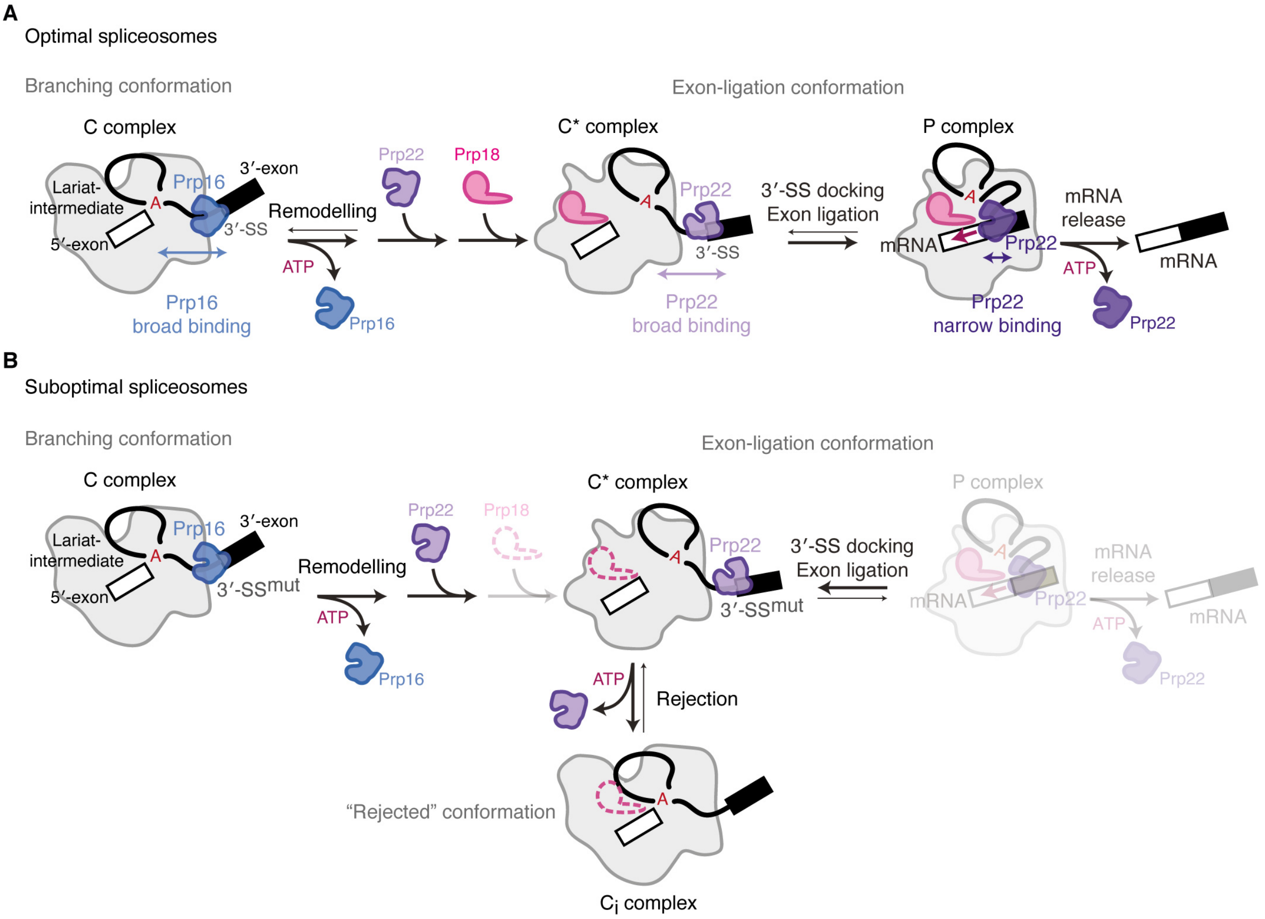

Supplementary Fig. 8 Model for substrate binding by spliceosomal helicases during the catalytic stage of pre-mRNA splicing in yeast. A Binding to optimal spliceosomes. After branching, Prp16 binds the lariat-intermediate broadly downstream of the brA, though the main binding peaks are observed between the brA and the 3′-SS. Prp16 dissociates during remodelling to the C* conformation, in which it is replaced by Prp22 before docking of the 3′-SS. Prp18 stabilises the exon-ligation conformation. In complex C*, before exon ligation, Prp22 binds the lariat-intermediate broadly on the 3′-exon and the intron. Such broad binding may facilitate 3′-SS docking to allow exon ligation. After exon ligation, in complex P, Prp22 engages the mRNA in a narrow binding window on the 3′-exon, from which it pulls on the RNA substrate to release the mRNA and dissociates following mRNA release. **B** Proofreading of suboptimal spliceosomes by Prp22. When the 3′-SS is non-canonical (3′-SS^{mut}), or in the absence of exon-ligation factors like Prp18, the exon-ligation conformation is destabilised. Prp22 binds along the 3′ exon and upstream of the 3′-SS on the intron, thus disfavouring stable 3′-SS docking, which prevents spliceosomes from catalysing exon ligation. Instead, Prp22 ATPase activity rejects these suboptimal C* complexes. As a result, Prp22 may completely or partially dissociate, thus further destabilising the exon-ligation conformation. Rejected spliceosomes may revert back to a C-like conformation (C_i), thus further minimising the potential for docking of the suboptimal 3′-SS.

Supplementary Tables

Supplementary Table 1. Yeast strains used in this study.

Strain	Resistance casette	Casette plasmids	Integration
SmB-3XFLAG-His8	TEF:nat:TEF	pAG25-3xFLAG-His8	genomic
Prp16-3XFLAG-His8	TEF:nat:TEF	pAG25-3xFLAG-His8	genomic
Prp22-3XFLAG-His8	TEF:nat:TEF	pAG-3xFLAG-His8	genomic
Prp22-3XFLAG-His8<br>Prp18-3XHA<br>Slu7-9Xcmyc	TEF:nat:ADH1<br>TEF:KanMX:TEF<br>TEF:hph:scCYC1	pBP83<br>pYM14<br>pYM20	genomic
Prp18-3XHA<br>Slu7-9Xcmyc	TEF:KanMX:TEF<br>TEF:hph:scCYC1	pYM14<br>pYM20	genomic
Prp16-G378A-CBP-3XFLAG-His6	URA, TRP		plasmid: pRS424, pRS426
CBP-Prp22-K512A-3XFLAG-His6	URA, TRP		plasmid: pRS424, pRS426

The first column indicates the modification compared to the parent strain BCY123. For strains with a genomic integration (indicated in the third), the resistance gene that was used for integration of the C-terminal tag is indicated with its promoter and terminator sequence, as well as the cassette plasmid used. Plasmids for over-expression of recombinant proteins are given in the fourth column together with the respective selection marker.

Supplementary Tables

Supplementary Table 2. pre-mRNA substrates used in this study.

Substrate	Exon 1	Intron distance (5′-SS to brA)	Intron distance (brA to 3′-SS)	Exon 2	Synthesis method	Label (3′ end)
3XMS2-UBC4-3′-UAc short	100 MS2 + 62	70	26	25	transcription + ligation	Cy5 (pre-labeled oligo)
3XMS2-UBC4	100 MS2 + 62	70	26	61	transcription + ligation	Cy5 (pre-labeled oligo)
3XMS2-UBC4-3′-UAC	100 MS2 + 62	70	26	61	transcription + ligation	Cy5 (pre-labeled oligo)
3XMS2-UBC4-3′-UAdG	100 MS2 + 62	70	26	61	transcription + ligation	Cy5 (pre-labeled oligo)
UBC4-3XMS2	20	70	26	20 + 100 MS2	transcription	Fluorescein (Cy2)
3XMS2-ACT1	100 MS2 + 32	266	44	161	transcription	pCp-Cy5 (enzymatic ligation)
3XMS2-ACT1-3′-UAC	100 MS2 + 32	266	44	161	transcription	pCp-Cy5 (enzymatic ligation)
3XMS2-UBC4-3′-UAdG	100 MS2 + 32	266	44	161	transcription + ligation	pCp-Cy5 (enzymatic ligation)
ACT1-3XMS2	25	266	44	161 + 100 MS2	transcription	pCp-Cy5 (enzymatic ligation)
3XMS2-ACT1-20	100 MS2 + 32	266	64	161	transcription	pCp-Cy5 (enzymatic ligation)
3XMS2-ACT1-40	100 MS2 + 32	266	84	161	transcription	pCp-Cy5 (enzymatic ligation)
3XMS2-ACT1-SL	100 MS2 + 32	266	84	161	transcription	pCp-Cy5 (enzymatic ligation)

The first column indicates the name of the transcript, including any modifications. The length of different transcript segments is indicated as number of nucleotides. Transcription + ligation indicates enzymatic ligation of a nucleotide pre-labelled with Cy5 during chemical synthesis.

Supplementary Tables

Supplementary Table 3. Oligonucleotides used in this study.

Number	Name	Sequence	Type
1	SmB_FLAG-His_a	GTTTAATAATGAAGCGCCCCCTCAAACAAGGAAGTTTCAGCCCCC ACCAGGTTTTAAAAGAAAAGACTACAAAGACCATGACGGTG	DNA
2	SmB_FLAG-His_b	CACATGCGTACACAAAAAAAGTATACGGAAACTATATTAGACTACA CTACATCAACCTTAGCGGCCGCATAGGCCACTAGTGG	DNA
3	Prp16_FLAG-His_a	GCAAAATATACTGAACGGCAAAGAAAATTCAATGAAACCTTTCAAA AGAAGGAAGCCTTTTTTTGACTACAAAGACCATGACGGTG	DNA
4	Prp16_FLAG-His_b	GCATGCATATAACTATATAATAACATATATGAATATTTTGCCTATTAGC ACGCTCTTCCCATAAAGCGGCCGCATAGGCCACTAGTGG	DNA
5	Prp22_FLAG-His_a	GGATCAAAATTCATGGAGACTAAGCTCAATAAGGCAGTCAAGGGA AAGGGCATTAGGTATCAAGAGGGACTACAAAGACCATGACGGTG	DNA
6	Prp22-FLAG-His_b	GTTGTTAAAAAATTAAATATAGGTCTATAAAACTCGATAATTATAATG CATAAAAAGCTAACAATGGCGGCCGCATAGGCCACTAGTGG	DNA
7	Prp22_FLAG_a	GTTAAAAAATTAAATATAGGTCTATAAAACTCGATAATTATAATGCATA AAAAGCTAACAATGTTAATCGATGAATTCGAGCTCGATTACAAC	DNA
8	Prp22_FLAG_b	CAAAATTCATGGAGACTAAGCTCAATAAGGCAGTCAAGGGAAAGG GCATTAGGTATCAAGAGGCGTACGCTGCAGGTCGACGACGAT	DNA
9	Prp18_HA_a	CCAGTATTAAAAGATTAATAACTTTTGAAGAATGGTATACCAGCAAC CACGATAGCTTAGCCCGTACGCTGCAGGTCGAC	DNA
10	Prp18_HA_b	CTTCTTATTTTGGCCGCATGATATCGTGCCACGCGATAACGAAAAC AATAGTTCAACAATTAATCGATGAATTCGAGCTCG	DNA
11	Slu7_cmyc_a	GGAAAGTAGATGGTACAAAGCAATCTGAGGAACAACGGAACCACT TAAAAGATTTATATGGTCGTACGCTGCAGGTCGAC	DNA
12	Slu7_cmyc_b	CTAAATTCTTTACCTCTCGTGCTCCGTGACTAGGATATTGGAAATT CAAATGATATAACTTAATCGATGAATTCGAGCTCG	DNA
13	Extension1a	CGATTTTATTTATTTGATCT	DNA
14	Extension1b	CGAGATCAAATAAATAAAAT	DNA
15	Extension2a	CGATTATTTATCTTGATTTT	DNA
16	Extension2b	CGAAAATCAAGATAAATAAT	DNA
17	dG-instert_ACT1	AUAUUAUAUGUUUA(dG)ACGUUGCUGCUUUGG	RNA with dG
18	Bridge_1_ACT1	GCAACGTCTAAACATATAATATGACAACAAAAAGAATGAAGC	DNA
19	Bridge_2_ACT1	CATACCAGAACCGTTATCAATAACCAAAGCAGC AACGTCTAAACATATA	DNA
20	dG-insert_UBC4	UAUUGAACUA(dG)ACAUCCACCUACUUCAUGUUCACGC	RNA with dG
21	AC-insert_UBC4	UAUUGAACUACACAUCCACCUACUUCAUGUUCACGC-Cy5*	RNA *with or without Cy5
22	AG-insert_UBC4	UAUUGAACUAGACAUCCACCUACUUCAUGUUCACGC	RNA
23	UBC4_extension	GGUCCACUCGGCGAUGAUCUAUAUCACUGGCAA GCA-Cy5	RNA with Cy5
24	Bridge_UBC4	AGATCATCGCCGAGTGGACCGCGTGAACATGAAGTAGGTGGATG TCTAGTTCAATAGCATGTTGAT TTTGTTAGTAAATA	DNA
25	RNase-H_UBC4	ATGAAGTAGGTGGAT	DNA
26	RNase-H_ACT1	AACCAAAGCAGCAAC	DNA

Sequences are given in the 5′ to 3′ direction. The last column indicates any additional modifications, such as Cy5-labelling. Oligonucleotides 1-12 were used for genetic modification of yeast strains. Oligonucleotides 13-16 were inserted to generate artificial *ACT1* transcripts. Oligonucleotides 17-24 were used to generate pre-mRNA substrates. Oligonucleotides 25-26 were used to induce RNase H digestion during P-complex purification.
