# Supplementary material for: psiCLIP reveals dynamic RNA binding by DEAH-box helicases before and after exon ligation
Source: Nat Commun. 2021 Mar 5;12:1488. doi: 10.1038/s41467-021-21745-9 (PMC7935899; doi:10.1038/s41467-021-21745-9)
Supplement: Supplementary file 4 — Description of additional supplementary files [file 41467_2021_21745_MOESM4_ESM.docx]

**Description of additional supplementary information**

**Title: Supplementary data 1**

**Description: Overview of psiCLIP samples included in this study.** The structure of the table reflects the deposited data found under E-MTAB-8895 (<https://www.ebi.ac.uk/arrayexpress/experiments/E-MTAB-8895/>) with the same sample names. Column B-H describe the experimental conditions stating the spliceosomal complex (H), pre-mRNA substrate (B, F), protein of interest (wt denotes for WT proteins, while dn denotes mutant proteins; C, D), and replicate number (E). Samples marked with FALSE in column G lack a FLAG-tagged protein and are therefore control samples. Column I indicates the Figures showing the analysis of the respective sample. Column J and K list the RT primer and adapter sequence used during the sample preparation, respectively.
